# First Year of Special Issue “New Insights in Vaccination and Public Health”: Opinions and Considerations

**DOI:** 10.3390/vaccines11030600

**Published:** 2023-03-06

**Authors:** Antonella Arghittu, Marco Dettori, Paolo Castiglia

**Affiliations:** 1 Department of Medicine, Surgery and Pharmacy, University of Sassari, 07100 Sassari, Italy; 2 University Hospital of Sassari, 07100 Sassari, Italy

## 1. Background

Disease prevention and control is the foundation of public health. In this respect, vaccinations have always been considered a means of proven efficacy, cost-effectiveness, and safety in the fight against infectious diseases [1]. Thanks to the legacy bequeathed by the first vaccine preparation by Edward Jenner in 1789, smallpox was eradicated, while other diseases (i.e., vaccine-preventable diseases—VPDs) are under control. Since then, millions of lives have been saved and countless cases of diseases and their complications have been prevented [2,3,4,5]. The COVID-19 pandemic further demonstrated how vaccine prophylaxis constitutes the main life-saving tool against VPDs [6].

In order to reach different sectors of the population and achieve better health, Public Health has made numerous efforts to empower citizens to make informed health choices (e.g., voluntary adherence to vaccination) while also making use of digital technologies. From the use of lifestyle monitoring systems to digital education, such as telemedicine, telerehabilitation, and self-medication and digital health interventions (e-health and m-health), information and communication technologies (ICTs) have become an integral component of care practices. In particular, m-health, with the use of digital tools such as websites, mobile applications, and social media [7,8,9], was widely used during the pandemic to facilitate care practices that were strongly conditioned by the health crisis [2].

Nevertheless, the issue of vaccine hesitancy continues to affect more than 15% of the world’s population, and even healthcare workers (HCWs) are expressing doubts about vaccination, with particular reference to the SARS-CoV-2 vaccine [10,11,12,13].

Moreover, the anti-vaccine content on the web has compounded the already precarious decision-making process, a dynamic conditioned by the traditional influence of social, cultural, political, and religious determinants on vaccine acceptance. This, due to a marked decrease in vaccination coverage, exposes the population to the risk of the re-emergence of infectious diseases that are today under control [14,15].

In such a broad context, the Special Issue “New Insights in Vaccination and Public Health”, published in the *Vaccines* journal (MDPI) on 1 October 2021, aimed to gather the best evidence on (i) the adoption of vaccination strategies implemented in order to increase vaccination coverage rates, (ii) the struggle against the issue of vaccine hesitancy, (iii) the implementation of health education interventions aimed at the general public and health care workers, and (iv) health communication and crisis communication interventions in a landscape where information and communication technologies have become part of individual and collective daily life. With this in mind, in its second year of publication, the Special Issue aims to identify some additional proven best practices for the implementation of decisive strategies to achieve the coveted health goals.

## 2. Manuscripts Published in the Special Issue

Reflecting upon the interest generated by the aforementioned topics of debate, as of 31 October 2022, 18 manuscripts have been accepted for publication in the Special Issue “New Insights in Vaccination and Public Health” following the peer review process. Specifically, 2 systematic reviews, 3 reviews, 11 articles, and 2 communications have been published online.

Several topics have been addressed, and all manuscripts that are published and available online in open-access form are listed in chronological order with respect to the date of publication in Table 1, indicating the following items: authorship (first author), topic, timing of the investigation, methodology, and main results of the study.

## 3. Discussion

Several topics were addressed in the Special Issue “New Insights in Vaccination and Public Health” in its first year of publication. Many of these explored the phenomenon of vaccine hesitancy [17,20,23,25,26,27,28,30,31,32] and, in particular, the acceptability of the COVID-19 vaccine [17,25,26,30,31] and the anti-influenza vaccine [23,28,33]. The role of ICTs was examined in relation to their influence on health choices [21,23,30] and in relation to the use of new technologies in the field of vaccinology [19]. Other topics, such as sex and gender differences in the response to the COVID-19 vaccine [16], serum prevalence surveys for varicella in pregnant women [18], mRNA COVID-19 vaccine reactogenicity among HCWs [22], the use of quantitative indicators in order to identify vaccine candidates [24], knowledge and behaviour regarding HPV infection and adherence to vaccine programs offered to adolescents [29], and the adoption of context-specific vaccine policies [32], were also addressed in this Special Issue.

Therefore, as aforementioned, in its second year of publication, this Special Issue aims to gather additional evidence for the implementation of adequate decisive vaccination strategies. Articles, systematic reviews or meta-analyses, brief communications and brief reports, letters or other types of articles dealing with the multidisciplinarity of these times and their impact on public health also with reference to different population cohorts (infants, adolescents, adults, the elderly, and at-risk populations) were welcomed and encouraged for this special sophomore issue. Manuscripts can be submitted to this Special Issue by 31 October 2023.

## Figures and Tables

**Table 1:** Description of the manuscripts accepted for the Special Issue “New Insights in Vaccination and Public Health” listed in chronological order of publication.

Authorship	Research Topic	Time Frame	Methodology	Main Findings
Heidari S. et al. [16]	Sex and gender differences in the response to COVID-19 vaccination	2019–2021	Systematic Review	The literature should further investigate sex and gender differences in the response to vaccination
Pal S. et al. [17]	Assessing vaccine hesitancy among US HCWs toward COVID-19 vaccination	2021	Cross-sectional survey	The hesitancy of some US HCWs to receive booster doses may reflect a general hesitancy to receive other forms of vaccination.
Lenis-Ballesteros V. et al. [18]	Estimating the seroprevalence of IgG for varicella in pregnant women and newborns	2016–2017	Cross-sectional study	High IgG seropositivity for VZV was detected in pregnant women, as was an efficient transfer of antibodies to the newborn. However, the proportion of unprotected pregnant women is significant.
Scognamiglio F. et al. [19]	New technologies in the field of vaccinology and Healthy Ageing	2022	Review	Advances in immunological knowledge and vacciniology highlight novel vaccination strategies adapted to target populations, such as the elderly and people with comorbidities.
Marzouk M. et al. [20]	Use of Monitoring and Evaluation frameworks and indicators for vaccination implementation	2022	Scoping Review	Minimal literature describing monitoring and evaluation frameworks or indicators for use in implementation of vaccination programs.
Olszowski R. et al. [21]	A Social Network Analysis of Tweets Related to Mandatory COVID-19 Vaccination	2021	Descriptive analysis	Substantial degree of polarisation, a high intensity of the discussion, and a high degree of involvement of Twitter users relating to mandatory COVID-19 vaccination in Poland.
Craciun M.D. et al. [22]	mRNA COVID-19 Vaccine Reactogenicity among HCWs	2021	Survey	Knowing the reactogenicity and safety profile of the administered vaccine can improve compliance and acceptance rate among healthcare professionals.
Yin H. et al. [23]	Investigation of factors influencing the flu and flu vaccination knowledge gap in the COVID-19 pandemic context	2022	Survey	There are gaps in knowledge about influenza and flu vaccination. However, internet media and social media are positively associated with knowledge levels.
Ma C. et al. [24]	Investigation of quantitative indicators in order to identify candidate vaccines to be included in the Expanded Immunization Program	2021	Delphi questionnaire	Using the resulting Delphi indicators, experts rank-ordered varicella, meningococcal conjugate AC, Hib, influenza, and EV71 vaccines for consideration of introduction into the EPI.
Waszkiewicz P. et al. [25]	Investigate the public acceptability of COVID-19 vaccination and actual levels of vaccination	2019–2020	Survey	The main factors that help increase vaccination acceptance are based on a self-centered pursuit of safety and freedom from restrictions.
Shkalim Zemer V. et al. [26]	To explore factors associated with low acceptance of the COVID-19 vaccine in adults in order to improve public health-targeted interventions in subpopulations with low vaccination rates	2021–2022	Observational study	Although COVID-19 vaccine uptake in the adult Israeli population is relatively high, it is significantly lower among minority groups and patients at high risk of severe course and complications of COVID-19, such as those with some comorbidities (asthma, smoking and diabetes mellitus).
Oduwole E.O. et al. [27]	To provide a broad overview of tools/measures addressing vaccine hesitancy	2010–2019	Review	Vaccine hesitancy can also be countered via the application of tools useful for its evaluation in high- and low-income countries.
Sallam M. et al. [28]	To investigate the possible association between influenza vaccine uptake/acceptance and the embrace of general vaccine conspiracy beliefs	2022	Cross-sectional survey	Contrasting vaccine hesitancy is essential to consider the psychological variables that influence the intention to receive flu vaccination.
Bencherit D. et al. [29]	To estimate knowledge about cervical cancer and HPV viruses, as well as to understand attitudes towards vaccination, both generally and related to HPV	2021–2022	Survey	Implementing social education activities and national HPV vaccination programs aimed at young people is strategic to the fight against HPV-induced cancers.
Aleksandric A. et al. [30]	To investigate vaccine hesitancy for COVID-19 vaccine in the Hispanic population by analysing social media data	2021	Descriptive analysis	Observation of social media can be a valuable tool for measuring attitudes toward public health interventions.
Motta M. [31]	To investigate any relationships and possible public health impact of vaccine hesitancy among people who received COVID-19 vaccine boosters in the United States	2022	Survey	Determinants such as convenience and confidence play a key role in the acceptance of the COVID-19 vaccine; therefore, they must be considered in the fight against vaccine hesitancy.
Mackenzie L-J. et al. [32]	To establish healthcare practitioners’ knowledge of vaccination practice and the preferred definition of shoulder injuries related to vaccine administration (SIRVA) between two available definitions	2022	Survey	Greater education and awareness of SIRVA and about SIRVA is needed in all healthcare disciplines.
Pi Z. et al. [33]	To examine whether public-funded influenza and PPSV23 immunization programs among elderly people in Beijing can be improved	2022	Cost-effectiveness analyses	Beijing’s current dual influenza and pneumococcal vaccination program was cost-effective among the elderly compared with the preceding policies of no vaccination and flu-only immunization programs
